# Chemistry-Assisted Proteomic Profiling of O-GlcNAcylation

**DOI:** 10.3389/fchem.2021.702260

**Published:** 2021-06-25

**Authors:** Qiang Zhu, Wen Yi

**Affiliations:** Department of Hepatobiliary and Pancreatic Surgery, Zhejiang Provincial Key Laboratory of Pancreatic Disease, The First Affiliated Hospital, School of Medicine, Zhejiang University, Hangzhou, China

**Keywords:** O-GlcNAcylation, enrichment strategies, mass spectrometry, quantitative proteomics, chemical tools

## Abstract

The modification on proteins with O-linked N-acetyl-β-D-glucosamine (O-GlcNAcylation) is essential for normal cell physiology. Dysregulation of O-GlcNAcylation leads to many human diseases, such as cancer, diabetes and neurodegenerative diseases. Recently, the functional role of O-GlcNAcylation in different physiological states has been elucidated due to the booming detection technologies. Chemical approaches for the enrichment of O-GlcNAcylated proteins combined with mass spectrometry-based proteomics enable the profiling of protein O-GlcNAcylation in a system-wide level. In this review, we summarize recent progresses on the enrichment and proteomic profiling of protein O-GlcNAcylation.

## Introduction

**FIGURE 1:**
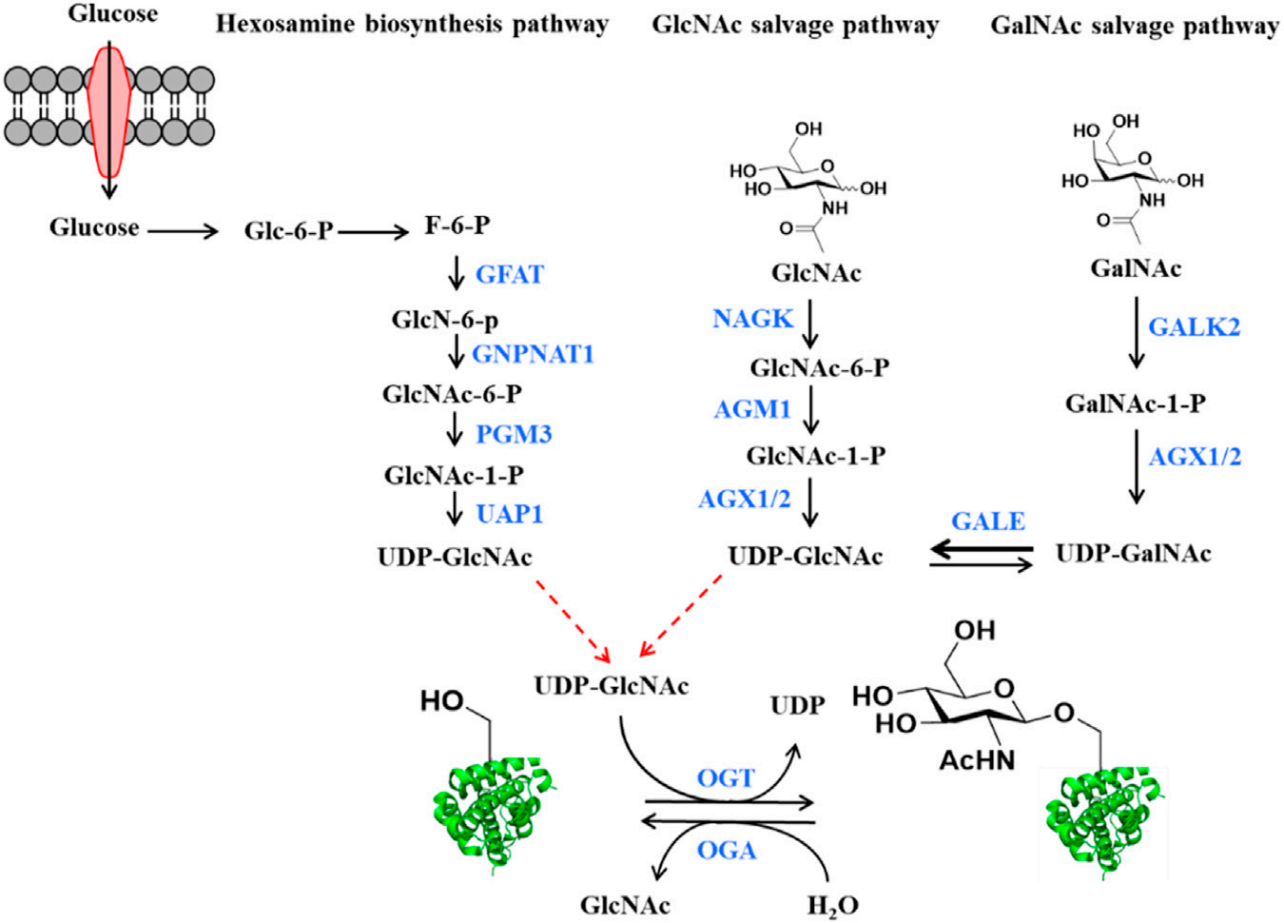
The source of O-GlcNAcylation donor UDP-GlcNAc in the cells. Glucose is converted to UDP-GlcNAc through the hexosamine biosynthetic pathway (HBP). Enzyme names shown in blue. GFAT: Glutamine fructose-6-phosphate amino-transferase, GNPNAT1: Glucosamine-Phosphate N-Acetyltransferase 1, PGM3: Phosphoglucomutase 3, UAP1: UDP-N-Acetylglucosamine pyrophosphorylase 1. UDP-GlcNAc also can be produced by the GlcNAc and GalNAc salvage pathways. Enzyme names shown in blue. NAGK: GlcNAc kinase, AGM1: GlcNAc-6-phosphate mutase, AGX1 or AGX2: UDP-GlcNAc pyrophosphorylase, GALK2: Galactokinase 2, GALE: UDP-galactose-4′-epimerase. Bold arrow represents that reaction is easier to perform. O-GlcNAc transferase (OGT) and O-GlcNAc hydrolase (OGA) regulate the addition and the removal of O-GlcNAc, respectively.

O-GlcNAcylation is a prevalent form of posttranslational modifications on the hydroxyl group of serine and/or threonine residues (Torres and Hart, 1984). Starting from fructose-6-phosphate, a glycolytic intermediate, a series of enzymatic reactions (collectively termed the hexosamine biosynthetic pathway) generate Uridine-Diphosphate N-acetylglucosamine (UDP-GlcNAc), the sugar donor for protein O-GlcNAcylation (Figure 1). UDP-GlcNAc can also be generated from the exogenous GlcNAc through the salvage pathway (Bond and Hanover, 2015), and by the enzymatic conversion of UDP-GalNAc by UDP-galactose-4′-epimerase (GALE) (Figure 1) (Boyce et al., 2011). Despite the occurrence of O-GlcNAcylation on numerous proteins, only two enzymes are responsible for the recycling of this modification in cells. O-GlcNAc transferase (OGT) catalyzes the addition of O-GlcNAc onto diverse protein substrates, while O-GlcNAc hydrolase (OGA) catalyzes the removal of this modification (Haltiwanger et al., 1990; Lubas et al., 1997). Notably, O-GlcNAcylation is reversible and highly dynamic in response to different cellular stimuli to regulate the structure and function of various intracellular proteins (Gao et al., 2001; Jang et al., 2012; Li and Yi, 2014; Ong et al., 2018). Besides, O-GlcNAcylation can interact with other posttranslational modifications including phosphorylation, acetylation and ubiquitination (Vercoutter-Edouart et al., 2015). These features make O-GlcNAcylation a regulator of various important and basic biological processes such as transcription, stem cell differentiation, signal transduction, cell cycle progression, and metabolic reprogramming (Hart et al., 2011; Bond and Hanover, 2015). For example, recent studies revealed that O-GlcNAcylation of Notch1 elevated its stability by abolishing the binding of E3 ubiquitin ligase Itch, thus maintaining the self-renewal of adult neural stem cells (Chen et al., 2021). Tan et al. found that O-GlcNAcylation of serine/arginine-rich protein kinase 2 (SRPK2) promoted *de novo* lipogenesis by regulating pre-mRNA splicing (Tan et al., 2021). Duan et al. revealed O-GlcNAcylation of RACK1 on serine 122 promoted its protein stability, ribosome binding and interaction with PKCβII to modulate hepatocellular carcinoma (HCC) tumorigenesis (Duan et al., 2018). Consequently, dysregulation of O-GlcNAc cycling has been implicated in the pathology of various diseases, including but not limited to, diabetes, cancer, cardiovascular diseases, and neuronal disorders (Slawson and Hart, 2011; Vaidyanathan and Wells, 2014; Yuzwa and Vocadlo, 2014). However, the specific molecular mechanisms by which O-GlcNAcylation contributes to the development and progression of these diseases remain to be elucidated.

Elucidating the function of O-GlcNAcylation in both physiological and pathological processes requires reliable and powerful detection tools to visualize and quantify the dynamics of O-GlcNAcylation. However, it is challenging to detect O-GlcNAcylated proteins by conventional techniques due to the regulatory nature of the modification (e.g., low abundance and highly dynamic) and the unique chemical characteristics (e.g., low immunogenicity and chemically/enzymatically labile) (Thompson et al., 2018). These features have called for the development of effective approaches to enrich and quantify this modification. In this review, we aim to provide a concise summary of recent advances to use chemistry-assisted proteomic methods to profile protein O-GlcNAcylation in a system-wise level.

## Enrichment Strategies for Protein O-GlcNAcylation

### Antibodies and Lectins

Unlike phosphorylation and other PTMs for which site-specific antibodies are available, effective and specific antibodies for O-GlcNAc are difficult to develop due to the low immunogenicity of the neutral O-GlcNAc sugar (Monsigny et al., 1979). The commonly used O-GlcNAc antibodies are two pan-antibodies (CTD110.6 and RL2), raised against glycopeptides derived from the C-terminal domain of RNA polymerase II, and rat liver nuclear envelopes, respectively (Snow et al., 1987; Comer et al., 2001). In addition, a few mouse monoclonal antibodies were developed, including HGAC85 (Turner et al., 1990), 10D8 (Yoshida et al., 1989), 18B10.7C (#3), 9D1. E4 (#10), and 1F5. D6 (#14) (Teo et al., 2010). These pan-antibodies were produced to yield the broad possible coverage of the modification. Although these antibodies can be employed for the detection of O-GlcNAcylated proteins, they exhibit different substrate recognition specificity. For example, CTD110.6, 18B10. 7C (#3), and 9D1. E4 (#10) are more inclined to recognize O-GlcNAc on the cell surface glycoproteins, and CTD110.6 shows cross-reactivity toward GlcNAc-containing N-glycans. RL2 also has a preference toward specific peptide sequences (Tashima and Stanley, 2014). In addition to the antibodies, specific lectins were also used in studies to detect O-GlcNAc. The lectin WGA (Wheat Germ Agglutinin) was first applied to detect and enrich O-GlcNAcylated proteins. But this plant lectin can recognize all terminal GlcNAc sugars as well as sialic acids (Monsigny et al., 1979; Snow et al., 1987). To increase the specificity, the succinyl WGA (sWGA) was developed, in which the recognition of sialic acid was inhibited via succinylation of WGA into the sialic acid recognition domain (Nakanuma et al., 1993). Another two fungal lectins PVL and AAL2 can bind to terminal non-reducing GlcNAc moieties (Kochibe and Matta, 1989; Ren et al., 2013). Recently, a recombinant lectin PVL (rPVL) produced from *Escherichia coli* was reported to have a higher specificity and affinity for proteins with multiple GlcNAc than WGA, AAL2 and PVLA (Machon et al., 2017). All of these lectins do not distinguish between terminal N-linked GlcNAc and O-GlcNAc residues, thus, the addition of PNGase F and/or sialidase is needed to remove the complex N-glycans and sialic acids during the detection and enrichment of O-GlcNAcylated proteins (Cieniewski-Bernard et al., 2004; Lefebvre et al., 2004; Zachara, 2009).

### Metabolic Labeling of O-GlcNAcylated Proteins

**FIGURE 2:**
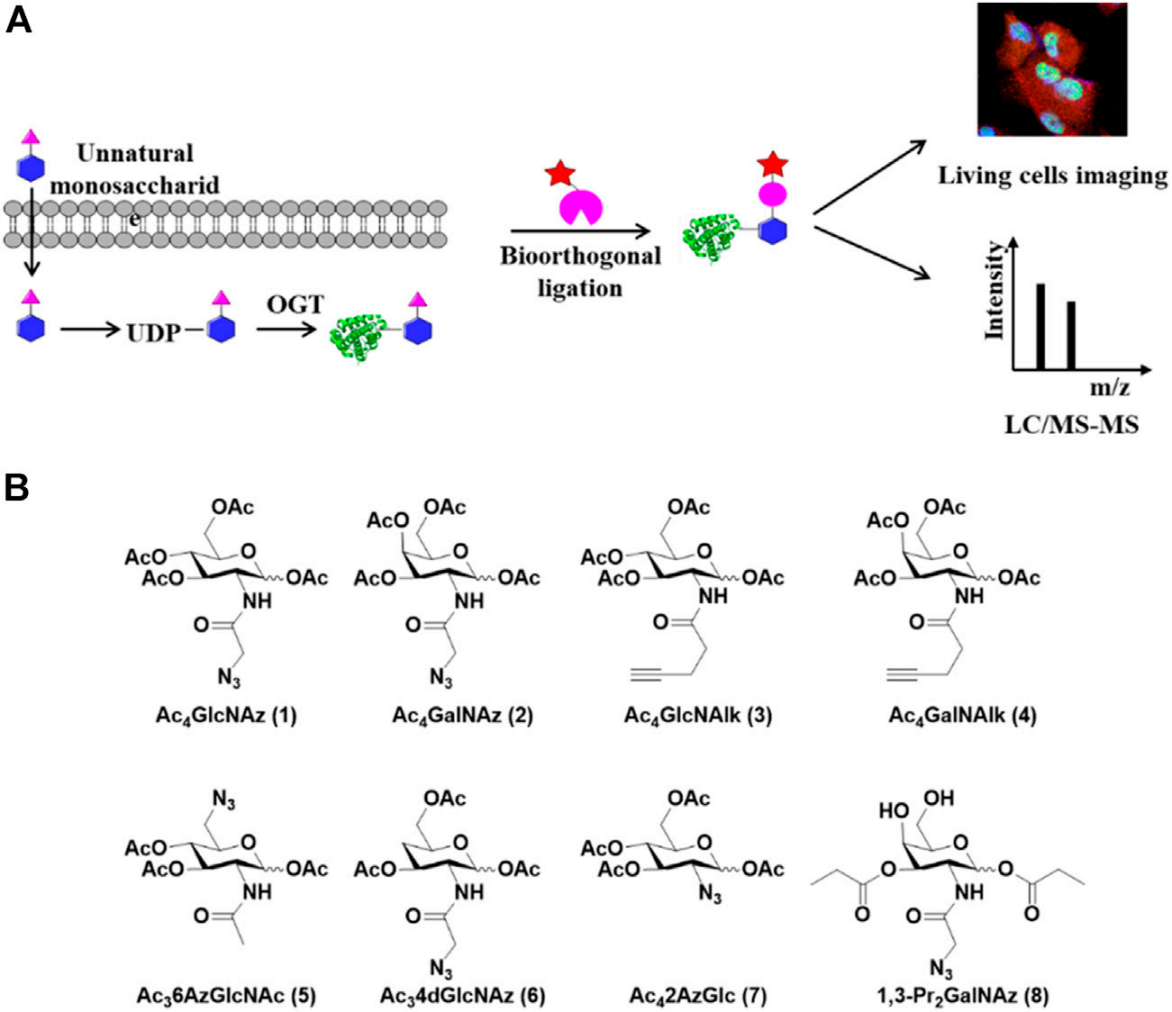
Metabolic labeling strategy for capture and detection of O-GlcNAcylated proteins. **(A)** Schematic of metabolic labeling. Unnatural monosaccharides enter cells and are metabolically converted to UDP-GlcNAc analogs which serve as donors for O-GlcNAcylation by OGT. The labeled O-GlcNAcylated proteins are enriched through bioorthogonal reactions and analyzed by LC-MS/MS or imaged in living cells. **(B)** Metabolic chemical reporters used for the labeling of O-GlcNAcylated proteins in cells.

Metabolic chemical reporters (MCRs) of glycosylation are unnatural monosaccharide analogs that contain bioorthogonal functionalities such as an alkyne or azide (Grammel and Hang, 2013; Chuh and Pratt, 2015; Chuh et al., 2016). Metabolic incorporation of these MCRs followed by bioorthogonal reactions such as the copper (I)-catalyzed azide-alkyne cycloaddition (CuAAC) has been extensively used to label complex glycans containing sialic acids, fucose, GalNAc, or GlcNAc (Figure 2A) (Prescher and Bertozzi, 2005; Best, 2009). In the detection of O-GlcNAcylation, a few specific MCRs were developed (Figure 2B). The first O-GlcNAc-targeted MCR 1,3,5,6-tetra-O-acetyl-N-azidoacetyl-glucosamine (Ac4GlcNAz, 1) has been employed for the visualization and proteomic profiling of O-GlcNAcylated proteins (Vocadlo et al., 2003). The acetyl groups act as protecting groups to enhance the permeability of GlcNAz into the cell. After deacetylation by cellular esterases, Ac4GlcNAz could be metabolically converted to UDP-GlcNAz which was then transferred to proteins by OGT (Zaro et al., 2011). Using this MCR, Hahne et al. identified about 1,500 O-GlcNAc proteins in cells. Coupled with β-elimination reaction, they mapped 185 O-GlcNAc modification sites on 80 proteins (Hahne et al., 2013). However, GlcNAz showed low selectivity since it could be incorporated into N-glycans (Cieniewski-Bernard et al., 2014). Subsequently, 1,3,5,6-tetra-O-acetyl-N-azidoacetyl-galactosamine (Ac_4_GalNAz, **2**) was used to label O-GlcNAcylated proteins (Boyce et al., 2011). However, further studies revealed that GlcNAz and GalNAz could interconvert to each other in cells, causing the labeling reaction unable to distinguish O-GlcNAc from mucin-type O-linked glycans (Chuh et al., 2014; Qin et al., 2020). To solve this problem, Zaro et al. (2011) employed alkyneacetyl-GlcNAc analogue (GlcNAlk, **3**) and alkyneacetyl-GalNAc analogue (GalNAlk, **4**) to label O-GlcNAcylated proteins. They found that GlcNAlk could not metabolically convert to GalNAlk, thus it would not label mucin-type O-linked glycans. GalNAlk showed a lower labeling efficiency since it was hard to metabolically convert to GlcNAlk in cells. Unfortunately, GlcNAlk was also incorporated into N-linked glycans, compromising the labeling specificity (Zaro et al., 2011). Chuh et al. (2014) circumvented this limitation by using 1,3,5-tri-O-acetyl-6-azido-6-deoxy-N-acetyl-glucosamine (Ac_3_6AzGlcNAc, **5**) (Chuh et al., 2014). Unlike the other MCRs mentioned above, 6AzGlcNAc could be directly phosphorylated at the 1-hydroxyl to bypass the canonical GlcNAc salvage pathway, which endowed it with a higher degree of selectivity for O-GlcNAcylated proteins (Chuh et al., 2014). However, 6AzGlcNAc showed a lower conversion efficiency to UDP-GlcNAc as compared to GlcNAz, emphasizing the potential balance between labeling efficiency and selectivity. Notably, O-GlcNAcylation is a dynamic process, in which the endogenous OGA can rapidly remove metabolic labels on proteins to decrease the labeling signal. Recently, hydrolysis-resistant MCRs such as 1, 3, 6-tri-O-acetyl-4-deoxy- N-azidoacetyl-glucosamine (Ac34dGlcNAz, **6**) and 1,3,5,6-tetra-O-acetyl-2-azido-2-deoxy-glucose (Ac_4_2AzGlc, **7**) have exhibited higher labeling efficiency and specificity for O-GlcNAc-modified proteins (Li et al., 2016; Zaro et al., 2017). Metabolic incorporation of Ac_4_2AzGlc is resistant to hydrolysis due to the lack of anchimeric assistance of the N-acetyl group (Macauley et al., 2005). Differently, Ac34dGlcNAz reduces nonspecific incorporation into extracellular glycans and increases resistance to OGA hydrolysis due to the absence of the 4′-OH group. (Cecioni and Vocadlo, 2013). Despite the enhancement of MCR permeability into cells by acetyl protecting groups, a recent study showed that per-O-acetylated azido and alkynyl sugars may spontaneously react with the cysteine side chains to generate S-glycosylation through a nonenzymatic mechanism (Qin et al., 2017). Therefore, per-O-acetylated sugar MCRs likely cause some false positives during the profiling of O-GlcNAcylation (Hao et al., 2019). On the other hand, the non-O-acetylated sugars are hard to cross the cell membrane for efficient labeling. To address this issue, 1,3-di-O-propionyl-N-azidoacetylgalactosamine (1,3-Pr2GalNAz, **8**) was developed as a novel metabolic probe for O-GlcNAc labeling which could be readily incorporated into O-GlcNAcylated proteins without introducing artificial S-glycosylation (Hao et al., 2019). Collectively, MCRs are robust and powerful tools to label and profile O-GlcNAcylated proteins in living cells.

### Chemoenzymatic Labeling of O-GlcNAc Proteins

**FIGURE 3:**
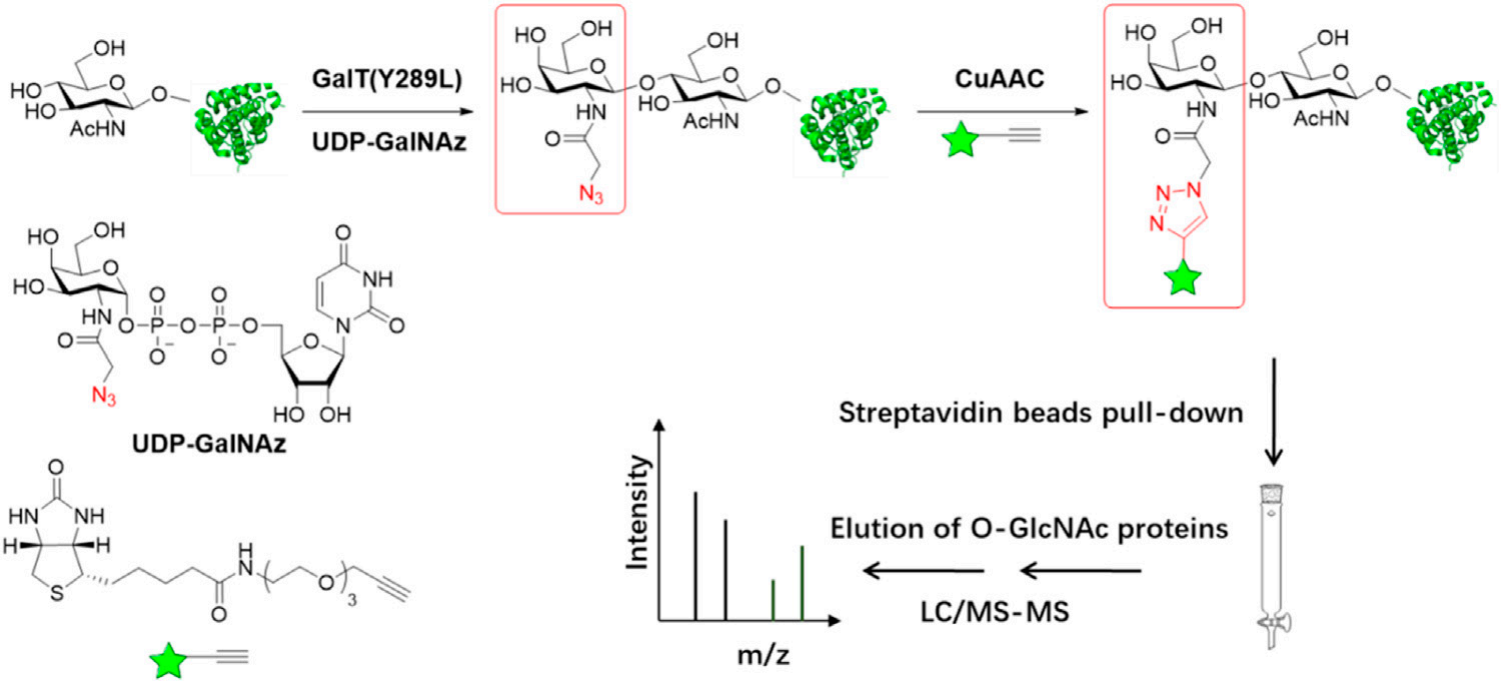
Schematic of chemoenzymatic labeling strategy for enrichment and detection of O-GlcNAc proteins. O-GlcNAcylated proteins in cell lysates are labeled with azide group using β-1,4-galactosyltransferase GalT (Y289L). The biotin with the alkyne group is added to O-GlcNAcylated proteins by copper(I)-catalyzed azide-alkyne cycloaddition (CuAAC) chemistry. Following labeling with biotin, O-GlcNAcylated proteins can be captured using streptavidin resin and then simultaneously proteins are digested and analyzed by LC-MS/MS.

As a complementary approach to the metabolic labeling, a chemoenzymatic labeling strategy has also been widely used in the capture and profiling of O-GlcNAcylated proteins (Figure 3). The Hart lab first developed a radioassay using a radiolabeled UDP-Gal and the enzyme β-1, 4-galactosyltransferase 1 (GalT1), which specifically transfers Gal to terminal GlcNAc moieties (Hayes et al., 1995; Torres and Hart, 1984). To extend the application of this method, a GalT1 mutant (Y289L) was generated to expand the substrate binding pocket, which allowed for the transfer of UDP-Gal analogues appended with chemical tags including azide, followed by bioorthogonal reactions, O-GlcNAc-modified peptides were biotinylated and subsequently were captured with avidin beads, eluted with free biotin, and sequenced by ETD mass spectrometry. (Figure 3) (Khidekel et al., 2003; Clark et al., 2008). Using this chemoenzymatic labeling strategy, the Hsieh-Wilson group carried out the first glycoproteomic study of O-GlcNAcylated proteins in the rat brain, in which some O-GlcNAc sites on 25 O-GlcNAcylated proteins were mapped (Khidekel et al., 2004). The bulky biotin group compromised the glycopeptide recovery efficiency. In the follow-up studies, a few cleavable enrichment probes were employed to improve the recovery of enriched glycopeptides and increase the rate of true assignment. The Hart group used a photocleavable biotin-alkyne probe to capture GalNAz-tagged O-GlcNAcylated peptides. When exposed to UV light (365 nm), O-GlcNAcylated peptides were released from the avidin chromatography column, followed by protein identification and site mapping by mass spectrometry (Wang et al., 2010a). Alfaro et al. used a photocleavable biotin probe to enrich and identify 274 O-GlcNAcylated proteins in mouse cerebral tissues (Alfaro et al., 2012). Tsumoto et al. used a novel alkyne probe containing thiol-alkyne to capture O-GlcNAcylated peptides. Glycopeptides were released by the reversible disulfide formation with a thiol-reactive resin (Tsumoto et al., 2015). Griffin et al. developed a probe that would be positively charged when it was cleaved to facilitate ETD-MS detection. An alkyne- 1-(4, 4-dimethyl-2, 6-dioxocyclohex-1-ylidene)ethyl (Dde)-biotin linker was used to label O-GlcNAcylated proteins. The Dde moiety can be quantitatively removed by hydrazine and showed higher labeling efficiency than PC-biotin-alkyne (Griffin et al., 2016). On the other hand, 3-ethynylbenzaldehyde probe was used to react with GalNAz via the copper-catalyzed Huisgen 1, 3-cycloaddition to form aromatic aldehyde-derivatized glycopeptides which were enriched by reversible hydrazone formation with hydrazide resins. Subsequently, glycopeptides could be eluted using hydroxylamine (Nishikaze et al., 2013).

In addition to capturing O-GlcNAcylated proteins, this strategy can be applied in live cell imaging, histological detection and modification stoichiometry quantification. Clark *el at*. labeled O-GlcNAcylated proteins selectively with a fluorescent reporter group to detect and image cellular O-GlcNAcylated proteins in living cells. (Clark et al., 2008). The Wu group applied this strategy to label histological specimens and demonstrated the change of O-GlcNAc levels during tumor development (Aguilar et al., 2017). To quantify O-GlcNAc stoichiometries on specific proteins, the Hsieh-Wilson group conjugated O-GlcNAcylated proteins with PEG mass tags. Compared to the nonglycosylated proteins, O-GlcNAcylated proteins showed the mass-shifted bands detected by immunoblotting with indicated antibodies. The occupancy levels of O-GlcNAcylation were determined by the intensity ratio of the glycosylated and nonglycosylated bands (Rexach et al., 2010). The Pratt group employed a semisynthetic O-GlcNAcylated protein standard combined with Strain-Promoted Cycloaddition (SPAAC) chemoenzymatic mass tagging protocol to improve the accuracy of O-GlcNAc stoichiometries analysis (Darabedian et al., 2018).

In summary, the biochemical tools and methods such as antibodies, lectins, metabolic, or chemoenzymatic labeling have different specificity and sensitivity in terms of enrichment and detection of O-GlcNAcylated proteins (Table 1). These strategies exhibit a broad range of applications including immunoblotting, proteomics, cellular and histological imaging (Table 1).

**TABLE 1:** The comparison of different detection methods for O-GlcNAcylation.

Detection methods	Specifficity	Sensitivity	Applications	References
Lectin	Low	Low	Western blot, proteomics	Vosseller et al. (2006), Chalkley et al. (2009)
Antibody	Moderate	Moderate	Western blot, proteomics	Snow et al. (1987), Teo et al. (2010), Comer et al. (2001)
Metabolic labeling	Moderate	High	Western blot, proteomics, live cell imaging, flow cytometry, in-gel fluorescence	Tan et al. (2018), Zaro et al. (2017), Qin W. et al. (2018), Li et al. (2016), Clark et al. (2008)
Chemoenzymatic labeling	High	High	Western blot, proteomics, in-gel fluorescence, histology, *in vivo* imaging	Rouhanifard et al. (2014), Aguilar et al. (2017), Li et al. (2019), Clark et al. (2008)

## Quantitative Proteomics for O-GlcNAcylation

O-GlcNAcylation is highly dynamic in response to various environmental stimuli. Quantifying its dynamics is key to elucidating the roles of O-GlcNAcylation in biological processes. Mass spectrometry-based quantitative proteomics, in combination with the aforementioned O-GlcNAcylated peptide enrichment methods, have recently emerged as a powerful tool to quantify protein O-GlcNAcylation in various biological settings.

### Stable Isotope Labeling With Amino Acids in Cell Culture-Based Quantitative Proteomics

**FIGURE 4:**
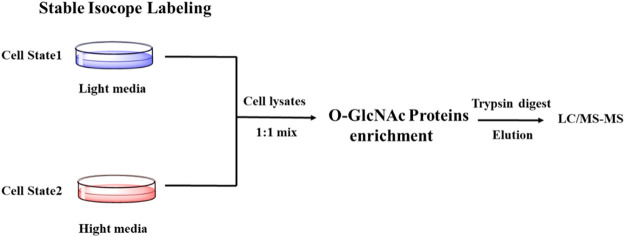
Schematic of SILAC-based Quantitative proteomics for profiling O-GlcNAcylation.

SILAC (Stable Isotope Labeling with Amino Acids in Cell Culture) is one of the most widely used quantitative proteomic techniques (Chen et al., 2015). Cells treated under different conditions are grown in the presence of normal (light) or isotopically enriched (heavy) versions of a specific label (amino acid, carbon, nitrogen) to produce unlabeled and fully labeled proteins. The glycosylated proteins are enriched, combined, followed by proteolysis and quantification by MS/MS. The mass shift due to the addition of isotope labeling in mass spectrometry can be used to quantify the difference in protein glycosylation abundance (Figure 4). Using SILAC-based quantitative proteomics, Zachara et al. identified 15 proteins that were dynamically modified by O-GlcNAc in response to heat stress (Zachara et al., 2011). Myers et al. found that occupancies of O-GlcNAc on different sites within the same protein were affected by polycombrepressive complex 2 (PRC2) in mouse embryonic stem cells, emphasizing the site-specific regulation of O-GlcNAcylation (Myers et al., 2011). The Hart group found that 10 proteins had an apparent increase of O-GlcNAcylation and 19 proteins showed a reduction of O-GlcNAcylation upon GSK-3 inhibition, indicating a complex interaction between phosphorylation and O-GlcNAcylation (Wang et al., 2007). Using SILAC combined with the chemoenzymatic labeling with a PC-biotin-alkyne tag, Wang et al. monitored the changes in the abundance of proteins and their O-GlcNAcylation during cytokinesis (Wang et al., 2010a). Recently, Qin et al. combined SILAC-based quantitative chemoproteomics with metabolic labeling using Ac36AzGlcNAc to analyze the turnover dynamics of O-GlcNAcylated proteins. Eventually, they identified 896 O-GlcNAcylated proteins, 86% of which showed a dynamic turnover in 12 h in the experiments (Qin et al., 2017).

### β-Elimination Followed by Michael Addition With Dithiothreitol-Based Quantitative Strategy

**FIGURE 5:**
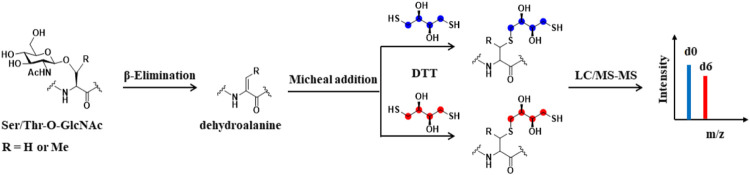
Schematic of BEMAD-based Quantitative strategy. O-GlcNAcylated serine and threonine residues can be converted to dehydroalanine by β-elimination reaction. Subsequently, isotope-labeled light DTT (DTTd0) or heavy DTT (DTT-d6) is added to O-GlcNAcylated peptides by Michael addition. The peptides are mixed and analyzed by LC-MS/MS. The red balls and blue balls represent heavy or light labeling sites, respectively.

Mapping O-GlcNAcylation sites is vital for elucidating the functional role of O-GlcNAcylation in specific biological environment. However, this can be very challenging because there is no known consensus sequence of O-GlcNAcylation on proteins, and that O-GlcNAc occurs in substoichiometry on many proteins. In addition, the O-glycosidic bond is labile, easily lost in collision-induced dissociation (CID) mass spectrometry (Greis et al., 1996; Whelan and Hart, 2006). To address these challenges, Hart and his colleagues developed the BEMAD method (β-elimination followed by Michael addition with dithiothreitol), which chemically converts O-GlcNAcylated serine and threonine residues into stable thiol derivatives (Figure 5) (Wells et al., 2002). Using the isotope-labeled dithiothreitol, BEMAD had been applied to quantify and map O-GlcNAcylation sites after chemoenzymatic labeling (Zachara et al., 2011; Tsumoto et al., 2017). The replacement of labile glycosylation with a more stable dithiothreitol modification significantly improved the efficiency of site-identification (Wells et al., 2002). The Hart group further employed isobaric tags for relative and absolute quantification (iTRAQ) and BEMAD coupled with the chemoenzymatic labeling to compare the site-specific O-GlcNAc occupancy on proteins obtained from normal and diabetic erythrocytes, highlighting the differentially regulated O-GlcNAcylation in diabetic erythrocytes (Wang et al., 2009). Sakabe et al. used the chemoenzymatic labeling and BEMAD method to identify various O-GlcNAc sites on histones H2A, H2B, and H4, elucidating that dynamic O-GlcNAcylation is a critical part of the histone code (Sakabe et al., 2009). Morover, Lund et al. employed a similar strategy to detect O-GlcNAc dynamics in response to T cell activation. More than 200 O-GlcNAcylated proteins were identified, among which are a number of proteins functionally related to RNA metabolism in human T cells, implying the functional importance of O-GlcNAcylation in T cell biology (Lund et al., 2016).

### QUIC and TMT Tag Strategies for Profiling O-GlcNAcylation

**FIGURE 6:**
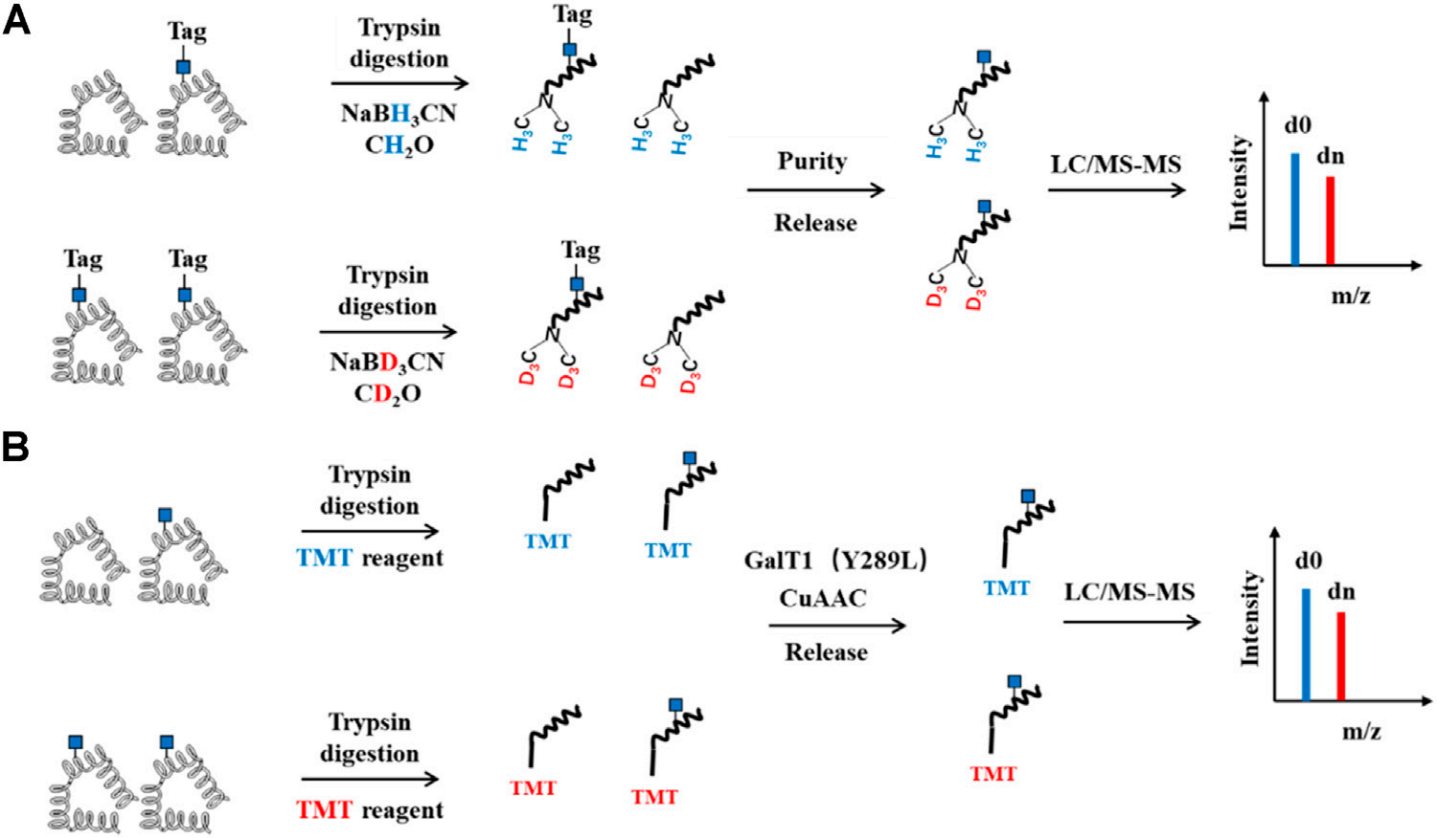
Schematic of QUIC and TMT Quantitative strategy for O-GlcNAcylation. **(A)** QUIC-Tag strategy. O-GlcNAcylated proteins first are chemoenzymatic labeled with a tag containing cleavable group and biotin, followed by trypsin digestion. The peptides are labeled with NaCNBH3 or NaCNBD3 by a modified dimethyl labeling strategy. Subsequently, O-GlcNAcylated peptides are enriched by streptavidin agarose and then released, mixed and analyzed by LC-MS/MS. **(B)** TMT strategy. The proteins are digested, labeled with TMT. O-GlcNAcylated peptides are then enriched, released and subjected to LC-MS/MS analysis.

Another approach couples O-GlcNAcylated peptide labeling/enrichment methods with tandem mass tagging for quantitative profiling of O-GlcNAcylation. The Hsieh-Wilson group developed a quantitative isotopic and chemoenzymatic tag (QUIC-Tag) strategy to identify and quantify O-GlcNAcylation in mouse brains in response to cellular stimulation. O-GlcNAcylated proteins were labeled selectively with a ketone-containing galactose analog via the chemoenzymatic strategy. The ketone functionality was reacted with an aminooxy biotin derivative, which can be captured by avidin chromatography. Subsequently, the proteins were digested and labeled with formaldehyde/NaCNBH3 or deuterated formaldehyde/NaCNBD3 by a modified dimethyl labeling strategy (Figure 6A). Coupled with tandem mass spectrometry, they demonstrated the dynamic O-GlcNAcylation in mediating neuronal communication (Khidekel et al., 2007). The tandem mass tag (TMT) probes containing an amine-reactive NHS-ester group, a spacer arm and an MS/MS reporter, are commonly used to label two to six peptide samples and measure relative protein expression levels with MS/MS (Thompson et al., 2003). Wang et al. integrated isobaric TMT labeling with chemoenzymatic enrichment to quantify O-GlcNAcylation between Alzheimer’s diseased brain and normal brain tissues. They identified 530 O-GlcNAcylated proteins covering 1,094 O-GlcNAcylation sites in the brain. The O-GlcNAcylation levels of 81 proteins in the Alzheimer's patients brain were changed, indicating that dysregulation of O-GlcNAcylation may play an important role in the development of Alzheimer’s disease (Wang et al., 2017).

### IsoTaG-Based Quantitative Proteomics

**FIGURE 7:**
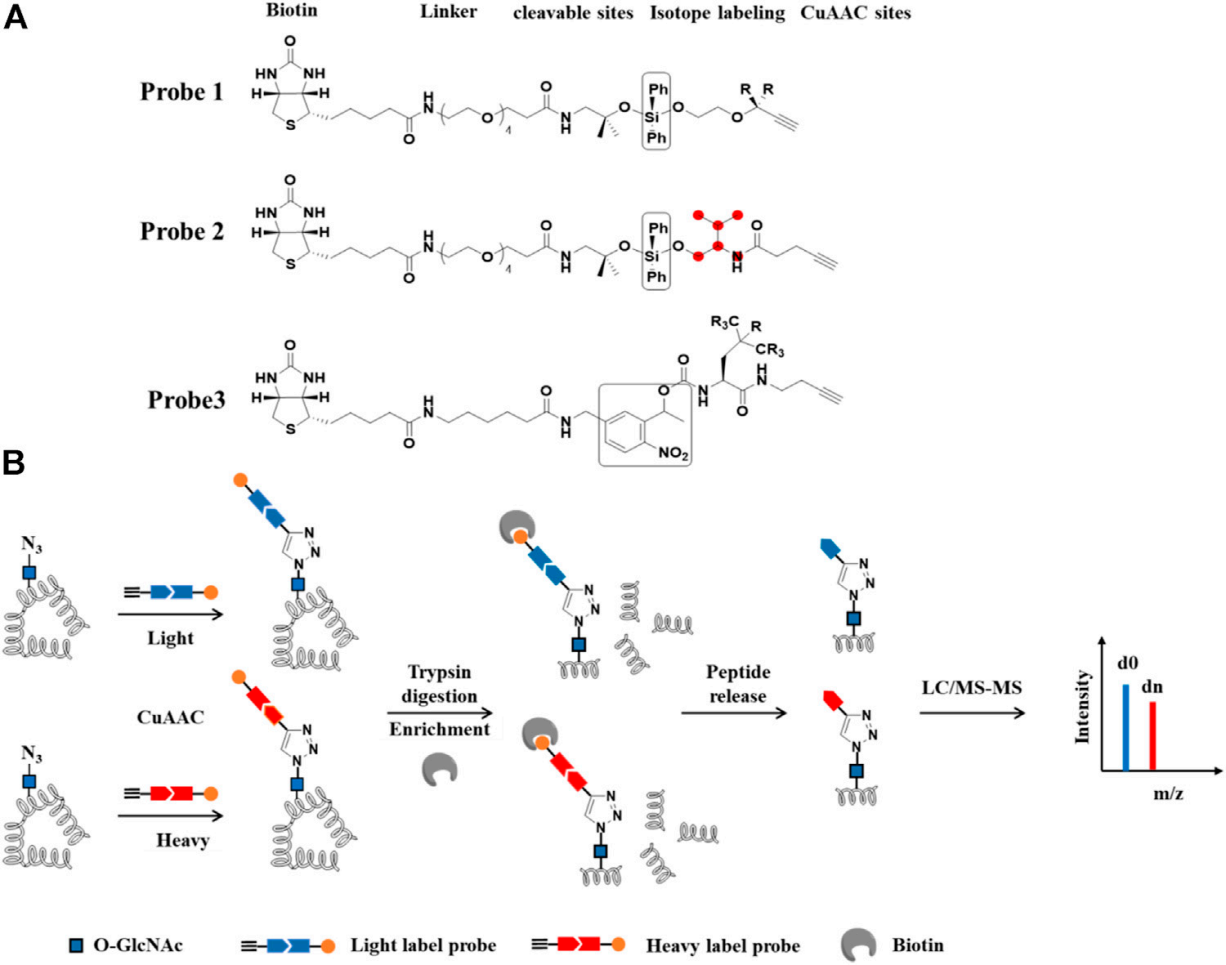
IsoTaG-based quantitative proteomics for profiling of protein O-GlcNAc modification. **(A)** Structures of probes. R and red balls represent heavy or light labeling sites. Black box represents cleavable groups **(B)** Workflow of differential labeling and quantitative analysis of O-GlcNAcylated proteins. O-GlcNAcylated proteins first are labeled with azide group by Metabolic labeling or chemoenzymatic strategies. Isotope probes are added to O-GlcNAcylated proteins via CuAAC reaction, followed by incubation with streptavidin beads. The proteins on the beads are digested with trypsin and O-GlcNAcylated peptides are released by cleavable sites and analyzed by LC-MS/MS.

Recently, an approach termed Isotope Targeted Glycoproteomics (IsoTaG) was developed by the Bertozzi group to enrich labeled glycopeptides and confidently profile the intact glycoproteome by MS (Woo et al., 2015). Specifically, glycoproteins were labeled metabolically with the azido functionality. Then, the soTaG silane Probe **1**, composed of an acid-cleavable biotin reagent containing an isotopic label and a terminal alkyne, was conjugated with the labeled glycoproteins. After capturing by streptavidin-agarose beads, and on-bead proteolytic digestion, the bound glycopeptides were released from the biotin tag and further sequenced by MS (Figure 7B). The quantification of the glycopeptides was achieved by using the pattern-searching algorithm mediated MS analysis to isotopically recoded species. IsoTaG shows a high sensitivity and repeatability when applied to low-abundance glycopeptides. Another strength of this method is that it promotes mass-independent targeted database searching for high-confidence distribution. With this strategy, Woo et al. metabolically labeled O-GlcNAcylated proteins with Ac_4_GalNAz to explore O-GlcNAcylation alterations in response to T-cell activation. They found that more than 500 glycopeptides underwent significant changes during T cell activation, facilitating the functional understanding of O-GlcNAcylation in resting and activated primary human T cells (Woo et al., 2018). Inspired by the IsoTaG strategy, Qin et al. developed an acid-cleavable dialkoxydiphenylsilane (DADPS) linker (Probe **2**) to quantify O-GlcNAcylation. The isotopically labeled DADPS probe could be used to capture glycopeptides, which were then released after cleavage with mild acid and quantified by comparing the isotopic ratios using ETD-based tandem mass spectroscopy (Qin K. et al., 2018). Similarly, Li et al. designed an isotope-coded photocleavable probe for quantifying O-GlcNAcylation. O-GlcNAcylated proteins from two different cell states were chemoenzymatically captured by the Probe **3**. The linker was cleaved when exposed to ultraviolet light (365 nm). The released glycopeptides were further analyzed for sites mapping and relative quantification (Li et al., 2019). They found that compared with sorafenib-sensitive liver carcinoma cells, 55 glycopeptides in the sorafenib-resistant cells showed an increase in O-GlcNAcylation stoichiometry, suggesting a role of O-GlcNAcylation in regulating tumor chemoresistance. Taken together, IsoTaG-based quantitative O-GlcNAcylation proteomics strategy greatly facilitates the quantification of glycoproteins by installing isotopic tags directly onto the O-GlcNAc moiety. The isotopic labeling can be used as the dual function to improve the reliability of glycopeptide assignment.

## Summary and Outlook

O-GlcNAcylation of intracellular proteins plays a fundamental role in health and disease. Effective ways to characterize the existence and dynamics of this modification will greatly promote the study of its functional significance. However, traditional methods, such as tritium labeling and the use of pan-O-GlcNAc antibodies, lack sensitivity and specificity. In addition, it is difficult to apply these methods to detect the changes and stoichiometry of O-GlcNAcylation in a complex system. The recent development of various chemical tools has provided exciting solutions to these problems. As stated above, a number of metabolic probes have been applied to detect and enrich O-GlcNAcylated proteins in living cells. A complementary chemoenzymatic labeling approach is also widely used to detect and profile O-GlcNAcylated proteins from cell lysates and tissues. Beyond the enrichment strategies, improvements in mass spectrometry technology have enabled quantifying and mapping O-GlcNAc sites with unprecedented accuracy.

O-GlcNAcylation is highly dynamic in response to nutrient availability and various environmental cues. With the variety of tools available to researchers, it is logical to profile and quantify O-GlcNAcylation under specific physiological contexts to reveal context-dependent functions of O-GlcNAc. In addition, current ways to modulate cellular O-GlcNAcylation rely on the use of small-molecule inhibitors or genetic knockdown/knockout, which lack the spatiotemporal resolution. Strategies that confer spatiotemporal control of O-GlcNAcylation are much needed. Moreover, O-GlcNAcylation on specific proteins has been shown to govern the protein function. Although there are some advances in developing strategies to manipulate protein-specific O-GlcNAcylation in cells (Gorelik and van Aalten, 2020; Ramirez et al., 2020; Ge et al., 2021), such studies are still in the infancy. In addition, the quantitative proteomics technique can't necessarily distinguish between changes in O-GlcNAc stoichiometry vs. changes in protein expression, which remains to be addressed. Nevertheless, we anticipate that further development of chemical tools will provide an important foundation for uncovering the functional importance of O-GlcNAcylation in the frontiers of biology and human health.
